# Loneliness and Mental Wellbeing in People With Inherited Macular Disease

**DOI:** 10.1007/s44402-026-00081-9

**Published:** 2026-04-15

**Authors:** Michael D. Crossland, Marc S. Tibber, Lottie A. G. Wood, Rachael F. Canavan, Tessa M. Dekker, Michel Michaelides

**Affiliations:** 1 UCL Institute of Ophthalmology, London, UK; 2 NIHR Moorfields Biomedical Research Centre, London, UK; 3 Research Department of Clinical, Education and Health Psychology, University College London, London, UK; 4 Experimental Psychology, Division of Psychology and Language Sciences, University College London, London, UK

**Keywords:** Inherited, Loneliness, Low vision, Macula, Vision impairment, Wellbeing

## Abstract

**Purpose:**

People with vision impairment have lower mental wellbeing than those without eye disease. Loneliness is thought to be associated with lower wellbeing in this population, but the confounding effect of mental ill-health and other factors which affect wellbeing has not been addressed. This study explored associations between loneliness and wellbeing in people with inherited macular disease, controlling for mental ill-health, demographic and clinical factors.

**Methods:**

Thirty-six people with inherited macular disease were recruited. Age, visual acuity, duration of disease and socioeconomic status were extracted from hospital notes. Loneliness, depression, anxiety and mental wellbeing were assessed using self-report questionnaires. Wellbeing was compared to a reference population of adults in the UK. Univariate and stepwise multivariate regression was used to examine the association between loneliness and wellbeing, controlling for demographic, clinical and mental health factors.

**Results:**

On average, participants’ wellbeing was not significantly different to the reference population (this study: mean: 50.9, sd: 9.68; reference population: mean = 51.4; sd: 9.42; ANOVA, *F* = 0.10, *p* = 0.75). Univariate linear regression showed significant associations between wellbeing and loneliness (*β* = −38.7; 95% CI: −57, −20; *p* < 0.0001), depression (*β* = −6.67; 95% CI: −10, −2.9; *p* < 0.0001) and anxiety (*β* = −5.37; 95% CI: −8.7, 2.0; *p* < 0.0001). None of the other demographic and clinical variables were related to wellbeing (all *p* > 0.05). Stepwise regression revealed loneliness, gender, depression and anxiety to be (together) significantly associated with wellbeing, accounting for 61% of the variance in wellbeing (*r*^2^ = 0.61).

**Conclusions:**

Loneliness was associated with lower mental wellbeing even after controlling for mental ill-health, demographic and clinical factors. The severity of vision loss cannot be taken as a simple index of likely wellbeing. Interventions which target loneliness may help maximise wellbeing in people with inherited macular disease.

Key Points

- Mental wellbeing for participants with inherited macular disease, who had access to low vision clinics and other support services, was similar to that of the general population.
- Participants reporting higher levels of loneliness had lower levels of mental wellbeing than those who were less lonely.
- This relationship persisted after controlling for mental ill-health, demographic or disease factors which could affect wellbeing.

## Introduction

Mental wellbeing is reduced in people with vision impairment [1, 2], but the reasons for this are not fully understood. Previous research has identified a variety of factors associated with poorer wellbeing in this population, including loneliness, lower social support, lower self-efficacy, unemployment and having a later onset of vision impairment [3].

Loneliness is strongly associated with reduced wellbeing, depression, anxiety, mental ill-health, poor physical health and increased mortality in the general population [4–6]. On average, people with vision impairment report higher levels of loneliness [7–10], and this is associated with lower life satisfaction [7] and depression [9]. Loneliness appears to be more likely in adults with vision impairment who live alone, who have limited mobility, who are not working or studying and who do not describe themselves as religious [7, 10]. This suggests loneliness is not a direct consequence of eye disease, but rather an indirect effect of the isolation and activity limitation which can be associated with vision impairment.

As well as having lower wellbeing, people with vision impairment are far more likely to experience depression [11] and anxiety [12] than their peers without sight loss. Although mental wellbeing is a broader concept than just the absence of mental ill-health, wellbeing is generally lower in people with depression or anxiety, which may confound previous research showing a relationship between loneliness and wellbeing in people with vision impairment.

To date, most research on wellbeing in people with eye disease has investigated older adults, with studies typically combining different causes of vision impairment, including (in some cases) cerebral vision impairment and syndromic conditions. However, different diseases may impact wellbeing in different ways. For example, the psychological impact of lifelong cerebral visual impairment is likely to be very different to having peripheral vision loss from glaucoma in later life, or central vision loss from inherited macular disease as a teenager. The wellbeing of adolescents with vision impairment is largely unexplored.

This paper explores the relationship between loneliness and mental wellbeing in a group of adolescents and adults with a single, non-syndromic, ocular cause of vision impairment: inherited macular disease. Demographic (age, sex, ethnic origin, socioeconomic status), clinical (visual acuity, duration of disease) and mental health (depression and anxiety) variables were controlled for, all of which are known to affect wellbeing. The primary hypothesis was that mental wellbeing would be lower in people with vision impairment who have higher levels of loneliness. The secondary hypothesis was that this effect would persist after the addition of other demographic, socioeconomic, clinical and mental health factors which may affect wellbeing. Finally, the predictive contributions of all of these variables of interest on mental wellbeing was examined, in order to identify those at greatest risk of reduced wellbeing.

## Methods

### Study Design

This was a cross-sectional observational study evaluating the wellbeing of people with vision impairment caused by inherited macular disease.

### Patient and Public Involvement and Engagement

The study was developed in collaboration with people with lived experience of vision impairment and their families, including representatives from the Stargardt’s Connected charity and the Moorfields Eye-Young Persons’ Advisory Group, who also reviewed the participant information documents.

One of the participants was asked to provide feedback on an early draft of this manuscript and evaluate the study with particular reference to the accessibility and ease of engagement in the study; their satisfaction with their participation in the study; and their comments on the clarity of the manuscript.

### Participants

People aged 13 years or older attending genetic eye disease or low vision clinics at Moorfields Eye Hospital who had a diagnosis of inherited macular disease were invited to participate. As assessed by the study team, those who did not have sufficient English language skills to complete the assessments, or who had hearing loss that affected their ability to complete telephone assessments, were excluded.

### Data Collection

Demographic details, including age, sex and ethnicity, were extracted from hospital records, as well as relevant medical details (type of inherited macular disease, time of diagnosis, time of symptom onset and visual acuity in the better eye). Age was categorised as adolescent (13–17 years), emerging adult (18–29 years), adult (30–64 years) or older adult (>64 years). Visual acuity was categorised as mild, moderate, severe or blind, in line with ICD-11 guidelines [13]. Participants’ home postcode using the 2019 English indices of deprivation, which links socioeconomic status to residents’ lower-layer super output area (a region of approximately 400–1200 households, or 1000–3000 people), was used as a proxy for socioeconomic status [14]. These indices were then categorised in deciles, with category 1 representing the most deprived 10% of English households and category 10 the least deprived 10%.

Loneliness, depression, anxiety and mental wellbeing were measured during a single telephone call between each participant and a member of the study team, at a time of the participant’s choosing.

Loneliness was measured using the UCLA (University of California, Los Angeles) loneliness scale version 3 [15] for adolescents (aged 13–17 years), and the revised UCLA loneliness scale [16] for those over 18 years. These instruments comprise 20 items, have four response levels and have high internal consistency, construct validity and test-retest correlation. Higher values on these scales relate to greater loneliness.

Depression was measured using the 25-item (four-response level) Revised Children’s Anxiety and Depression Scale (RCADS-25 [17]) in adolescents and the nine-item, four-response level, Patient Health Questionnaire 9 (PHQ-9 [18]) in adults. Anxiety was measured using the RCADS-25 in adolescents and the Generalized Anxiety Disorder-7 (GAD-7 [19]) seven-item, four-response level instrument in adults. On these scales, higher values relate to higher levels of depression and anxiety.

Mental wellbeing was measured with the Warwick-Edinburgh Mental Wellbeing Scale (WEMWS) for all participants [20]. On this scale, higher values correspond to  better wellbeing, respectively. All have been well validated and show robust psychometric properties [18–20].

At the end of the data collection session, participants were offered referral to age-appropriate counselling services. A suicide and self-harm risk assessment protocol was in place, developed by a senior clinical psychologist (author MST). All members of the research team were offered clinical supervision and had access to confidential counselling services.

### Statistical Design

Data were collected using the secure Research Electronic Data Capture (REDCap; project-redcap.org) system hosted by University College London and stored in a secure database. All data were tested for normality using the Shapiro–Wilk test, with log transformations being performed where data departed from normality at the *p* < 0.05 level.

Analysis of variance (ANOVA) was used to compare wellbeing values to benchmark data from the Health Survey for England [21]. Descriptive statistics were used to identify caseness for depression and anxiety for included participants. Caseness is a term widely used in psychiatry which refers to meeting the diagnostic threshold for a disorder. RCADS-25 data were converted to T-scores, normalised by age and gender, for depression and anxiety, based on Ebesutani’s dataset [22]. T-scores <65 were defined as in the ‘normal range’, between 65 and 69 as in the ‘borderline clinical range’ and ≥70 as in the ‘clinical range’. For adults, following clinical recommendations, caseness for depression and anxiety were defined as scores of ≥10 on the PHQ-9 [18] and GAD-7 [19], respectively. This allowed for comparison between adolescents and adults.

To explore the relationship between wellbeing and loneliness, several linear regression analyses were performed. First, a series of univariate regression analyses were undertaken, regressing wellbeing (separately) on loneliness and covariates including demographic (age, gender, ethnic origin, socioeconomic status), clinical (visual acuity, duration of disease) and mental health (caseness for depression and anxiety) variables to explore basic patterns of association. Next, two additional models were run, using forward stepwise multivariate regression with a relatively liberal probability level for entry of 0.15, to avoid excluding important variables [23]. In the first, wellbeing was regressed on loneliness, with demographic and clinical variables assessed for inclusion; in the second, the same model was run, with mental health variables also assessed for inclusion.

Analyses were performed in JMP Pro (v. 18.2.2, JMP Statistical Discovery LLC, jmp.com).

## Results

Thirty-six participants were recruited. Participants’ demographic, socioeconomic, clinical and mental health characteristics are shown in Table 1. Duration of disease and loneliness data were positively skewed and were log-transformed.

**Table 1:** Participant demographics and clinical characteristics.

		Number (%)
Age group	Adolescent (13–17 years)	9 (25%)
	Emerging adult (18–29 years)	6 (17%)
	Adult (30–64 years)	16 (44%)
	Older adult (>64 years)	5 (24%)
Gender	Female	17 (47%)
	Male	19 (53%)
Ethnic group	Asian or Asian British	13 (36%)
	Mixed or multiple ethnic groups	6 (17%)
	White	13 (36%)
	Not stated	4 (11%)
Socioeconomic status (decile)	1 (most deprived)	7 (21%)
	2	2 (6%)
	3	2 (6%)
	4	1 (3%)
	5	4 (12%)
	6	4 (12%)
	7	6 (12%)
	8	4 (12%)
	9	3 (9%)
	10 (least deprived)	1 (3%)
	Missing data	2 (6%)
Disease	ABCA4/Stargardt	20 (56%)
	Best disease	3 (8%)
	Other IMD	9 (25%)
	Genetically unconfirmed	4 (11%)
Vision impairment category	Not vision impaired	2 (5.6%)
	Mild	7 (19%)
	Moderate	17 (47%)
	Severe	7 (19%)
	Blind	5 (14%)
Anxiety caseness	Yes	9 (25%)
	No	27 (75%)
Depression caseness	Yes	6 (17%)
	No	30 (83%)
		**Median (25th, 75th centile)**
Duration of disease	Years	11 (5, 30)
Loneliness	UCLA scale	31 (26, 41)

Socioeconomic status is based on home postcode.

The vision impairment category is based on the International Classification of Diseases (ICD-11) criteria. Anxiety caseness is defined by RCADS-25 in adolescents and GAD-7 in adults. Depression caseness is defined by RCADS-25 in adolescents and PHQ-9 in adults.

*IMD* inherited macular disease, *UCLA* University of California, Los Angeles loneliness scale.

The mean wellbeing score, measured on the WEMWS, was 50.9 (sd: 9.7). On average, participants’ wellbeing was not significantly different to that of the general population (general population; mean = 51.4; sd: 9.42; this study: mean: 50.9, sd: 9.68; ANOVA, *F* = 0.10, *p* = 0.75). Ten of the participants had ‘moderate’ levels of loneliness and five had ‘moderate/high’ loneliness.

Univariate linear regression showed a significant linear association between wellbeing and loneliness (*r* = −0.59, *p* < 0.001). With respect to other variables explored, wellbeing was significantly associated with depression (*r*^2^ = 0.27, *p* < 0.01) and anxiety (*r*^2^ = 0.24, *p* < 0.01) (Table 2). None of the demographic or clinical variables, including severity of vision loss, were related to wellbeing (*p* > 0.05). Loneliness was not correlated with having depression (*χ*^2^ = 0.002, *p* = 0.96) or anxiety (*χ*^2^ = 1.13, *p* = 0.29).

**Table 2:** Results of univariate and multivariate regression analyses of wellbeing.

		Univariate models	Multivariate model with demographic and clinical variables	Multivariate model with demographic, clinical and mental ill-health variables
	Category	Coefficient (95% CI)	Coefficient (95% CI)	Coefficient (95% CI)
Loneliness (log UCLA score)	**-**	**−38.7 (−57, −20)** ***p*** < **0.001**	**−37.2 (−54, −20)** ***p*** < **0.001**	**−35.7 (−51, −20)** ***p*** < **0.0001**
Age group (reference level: adolescent)	Emerging adult	2.19 (−2.6, 7.0) *p* = 0.34		
	Adult	1.14 (−3.5, 5.8) *p* = 0.62		
	Older adult	5.10 (−0.11, 10) *p* = 0.05		
Gender	Male	2.85 (−0.21, 5.9) *p* = 0.08	**−3.18 (−5.5, −0.83)** ***p*** < **0.01**	−1.83 (−4.1, 0.47) *p* = 0.12
Ethnic group (reference level: white British)	All not white British	1.90 (−1.79, 5.58) *p* = 0.30		
Socioeconomic status		−0.25 (−1.45, 0.95) *p* = 0.68		
Vision impairment level (reference level: none)	Mild	6.10 (−4.0, 16) *p* = 0.18		
	Moderate	2.70 (−4.9, 10) *p* = 0.47		
	Severe	6.07 (−0.9, 13) *p* = 0.08		
	Blind	1.30 (−7.5, 10) *p* = 0.72		
Duration of disease (log years)	-	−2.39 (−9.2, 4.5) *p* = 0.50		
Depression	Yes	**−6.67 (−10, −2.9)** ***p*** < **0.01**		**−3.76 (−7.3, −0.26)** ***p*** < **0.05**
Anxiety	Yes	**−5.37 (−8.7, 2.0)** ***p*** < **0.01**		−2.05 (−4.8, 0.67) *p* = 0.14

Results show model regression coefficients. For categorical variables, reference levels are given. Bold values show statistically significant associations.

*UCLA* University of California, Los Angeles loneliness scale, *CI* confidence interval.

In the first stepwise regression (in which demographic, socioeconomic and clinical variables were added), the association between wellbeing and loneliness remained significant (*r*^*2*^ = 0.31, *p* < 0.01) and gender was retained (*r*^*2*^ = 0.43, *p* < 0.05); overall, the model accounted for 47% of the variance in wellbeing (*r*^2^ = 0.47). In the second stepwise regression model (in which anxiety and depression were also added), the association between wellbeing remained significant (*r*^*2*^ = 0.27, *p* < 0.01), and gender, depression and anxiety were retained; overall, the model accounted for 61% of the variance in wellbeing (*r*^2^ = 0.61).

### Participant Review

The participant confirmed that the study asked an important research question, used appropriate methods and recruited an appropriate population. He did not experience any accessibility problems with the study and did not find any of the questions ambiguous. He made some comments on the readability of the manuscript (specifically, the use of acronyms), which have been incorporated.

## Discussion

As hypothesised, an association was found between loneliness and mental wellbeing in adolescents and adults with inherited eye disease, with people reporting higher levels of loneliness also indicating lower levels of wellbeing. This supports the findings of other research linking loneliness with wellbeing in eye disease [7–10], and extends these findings by showing that this relationship remained significant after controlling for demographic, socioeconomic, clinical and mental health variables. Depression and anxiety were also associated with lower levels of wellbeing, but demographic and clinical factors were not.

Levels of wellbeing experienced by the participants were broadly similar to those of the general population. The discrepancy between this finding and previous work, which showed lower levels of wellbeing in people with vision impairment relative to the general population [1–3], might be due to the current participants being recruited from a specialist ophthalmology centre, where they had access to low vision assessment, counselling, emotional and genetic counselling, eye clinic liaison officers and expert ophthalmology care. Additionally, participation biases may have excluded those with the lowest levels of wellbeing.

Low mental wellbeing is known to be a risk factor for the development of major depression [24]. Encouragingly, loneliness appears to be potentially modifiable, for example, by social prescribing [25] and mentoring programmes [26]. A particularly encouraging finding of the present work is that there was no association between wellbeing and *nonmodifiable* risk factors, such as age, ethnicity, disease severity and disease duration. The severity of vision loss cannot be taken as a simple index of wellbeing or mental ill-health risk; instead, the relationship between eye disease and wellbeing is likely to be more complex and dependent on multiple social and environmental factors.

Unlike Verstraten et al. [9], who measured loneliness and depression in a cohort of older adults, the present study did not find a relationship between depression and loneliness. The reason for this apparent discrepancy is unclear, though the lower and broader age distribution of the current sample is one possibility. For example, the effects of loneliness may be elevated in older populations, who commonly experience greater levels of social isolation [27, 28].

Would depression and loneliness affect wellbeing to the same extent in people *without* vision impairment? Perhaps, but as depression [11] and loneliness [7, 9] are both higher in people with vision impairment, it appears that the impact of these factors on wellbeing in this population is particularly important. Interventions to target loneliness (such as social prescribing, support groups and befriending services) and depression (such as counselling and prompt referral to mental health services) should be tailored and offered to people with vision impairment and evaluated empirically.

### Limitations

The biggest limitation of the present study is its relatively small sample size, largely caused by the relative rarity of inherited macular disease and the exploratory nature of this research. This may have reduced the statistical power of the findings. A further limitation was that the study was cross-sectional in nature, so that causation, or the underlying direction thereof, could not be inferred. However, we are in the process of collecting longitudinal data from the adolescent participants in this study.

As discussed above, the present participants may not be fully representative of the general population with inherited macular disease. People with vision impairment who are medically excluded, or those who do not have access to a specialist centre with such a comprehensive support service, may have lower wellbeing when compared to people included in this study. All of the participants had ‘low,’ ‘moderate’ or ‘moderate/high’ levels of loneliness, with none having the highest level of loneliness as measured on the UCLA scale. Additionally, all of the participants received services through the National Health Service, free of charge at the point of delivery, which may limit the applicability of the results to non-European settings.

Although an impact of socioeconomic status on mental health was not found, people from areas of higher social deprivation are less likely to access eyecare [29] and may not have been recruited for the present study. Further, the method of using an area-level proxy for socioeconomic status is relatively unreliable.

Future research should consider including a larger population of people with vision impairment, including those who are medically excluded or who have not received multidisciplinary support. A more detailed exploration of the role of socioeconomic status, using more rigorous methods, would also be of interest.

## Conclusions

Loneliness was found to be strongly associated with reduced wellbeing in a group of adolescents and adults with vision impairment caused by inherited macular disease. Disease factors, such as the severity of vision loss, cannot be used as a proxy or a predictor of likely wellbeing. The role of interventions which support loneliness, such as social prescribing and mentoring, should be explored for people with vision impairment.

## Data Availability

The datasets used and/or analysed during the current study are available from the corresponding author on reasonable request.
